# Unlocking *lox*P to Track Genome Editing In Vivo

**DOI:** 10.3390/genes12081204

**Published:** 2021-08-03

**Authors:** William A. C. Gendron, Jeffrey D. Rubin, Michael J. Hansen, Rebecca A. Nace, Brandon W. Simone, Stephen C. Ekker, Michael A. Barry

**Affiliations:** 1 Virology and Gene Therapy Graduate Program, Mayo Clinic Graduate School of Biomedical Sciences, Rochester, MN 55905, USA; gendron.william@mayo.edu (W.A.C.G.); rubin.jeffrey@mayo.edu (J.D.R.); hansen.michael@mayo.edu (M.J.H.); nace.rebecca@mayo.edu (R.A.N.); 2 Department of Biochemistry and Molecular Biology, Mayo Clinic, Rochester, MN 55905, USA; simone.brandon@mayo.edu (B.W.S.); ekker.stephen@mayo.edu (S.C.E.); 3 Department of Medicine, Division of Infectious Diseases, Mayo Clinic, Rochester, MN 55905, USA; 4 Department of Immunology, Mayo Clinic, Rochester, MN 55905, USA; 5 Department of Molecular Medicine, Mayo Clinic, Rochester, MN 55905, USA

**Keywords:** CRISPR, SaCas9, ErCas12a, *lox*P, Cre, luciferase reporter, fluorescent reporter, gene editing, naked DNA injection, in vivo

## Abstract

The development of CRISPR-associated proteins, such as Cas9, has led to increased accessibility and ease of use in genome editing. However, additional tools are needed to quantify and identify successful genome editing events in living animals. We developed a method to rapidly quantify and monitor gene editing activity non-invasively in living animals that also facilitates confocal microscopy and nucleotide level analyses. Here we report a new CRISPR “fingerprinting” approach to activating luciferase and fluorescent proteins in mice as a function of gene editing. This system is based on experience with our prior cre recombinase (cre)-detector system and is designed for Cas editors able to target *lox*P including gRNAs for SaCas9 and ErCas12a. These CRISPRs cut specifically within *lox*P, an approach that is a departure from previous gene editing in vivo activity detection techniques that targeted adjacent stop sequences. In this sensor paradigm, CRISPR activity was monitored non-invasively in living cre reporter mice (FVB.129S6(B6)-Gt(ROSA)26Sortm1(Luc)Kael/J and Gt(ROSA)26Sortm4(ACTB-tdTomato,-EGFP)Luo/J, which will be referred to as LSL-luciferase and mT/mG throughout the paper) after intramuscular or intravenous hydrodynamic plasmid injections, demonstrating utility in two diverse organ systems. The same genome-editing event was examined at the cellular level in specific tissues by confocal microscopy to determine the identity and frequency of successfully genome-edited cells. Further, SaCas9 induced targeted editing at efficiencies that were comparable to cre, demonstrating high effective delivery and activity in a whole animal. This work establishes genome editing tools and models to track CRISPR editing in vivo non-invasively and to fingerprint the identity of targeted cells. This approach also enables similar utility for any of the thousands of previously generated *lox*P animal models.

## 1. Introduction

CRISPR-Cas technology has revolutionized the gene editing space, enabling access to the field for many new users [1]. Due to its versatility and ease of use, it is being deployed for usage in vivo as a method to treat genetic disorders requiring novel delivery methods and a means to monitor gene editing in vivo [2]. For example, somatic in vivo CRISPR–Cas9 gene editing was recently performed in humans as a treatment for Leiber’s congenital amaurosis 10 (LCA10) [3].

Previous methods to monitor in vivo CRISPR editing have commonly relied on nucleotide sequencing of edited target biopsies or invasive monitoring of the tissues for fluorescent markers [4]. These methods require the destruction of the tissue of interest and prevent long term serial monitoring of CRISPR editing. Additionally, without non-invasive monitoring, unpredicted off-target tissue activity may not be detected, unless each tissue is tested directly. Therefore, a method with the ability to monitor CRISPR activity non-invasively is an important tool for developing gene editing delivery systems.

The cre-*lox*P system is also a powerful tool for animal and cell models, providing robust and specific editing of DNA [5]. Cre recombinase is derived from bacteriophage P1. Cre recombines DNA contained within *lox*P sequences, creating inversions, deletions, and insertions depending on the orientation of the *lox*P sites [6]. Thousands of animal models have been generated using cre-*lox*P technologies, 320 of which are specifically mouse reporter models [7]. Many cre-*lox*P animal models have also been developed in which fluorescent or luminescent reporter genes are inactivated by upstream “floxed” sequences, which are two *lox*P sites surrounding a polyadenylation (polyA) “stop” sequence or another gene [8,9]. When the cells of these animals are exposed to cre, the floxed sequence is excised, this activates reporter gene expression.

**Figure 1:**
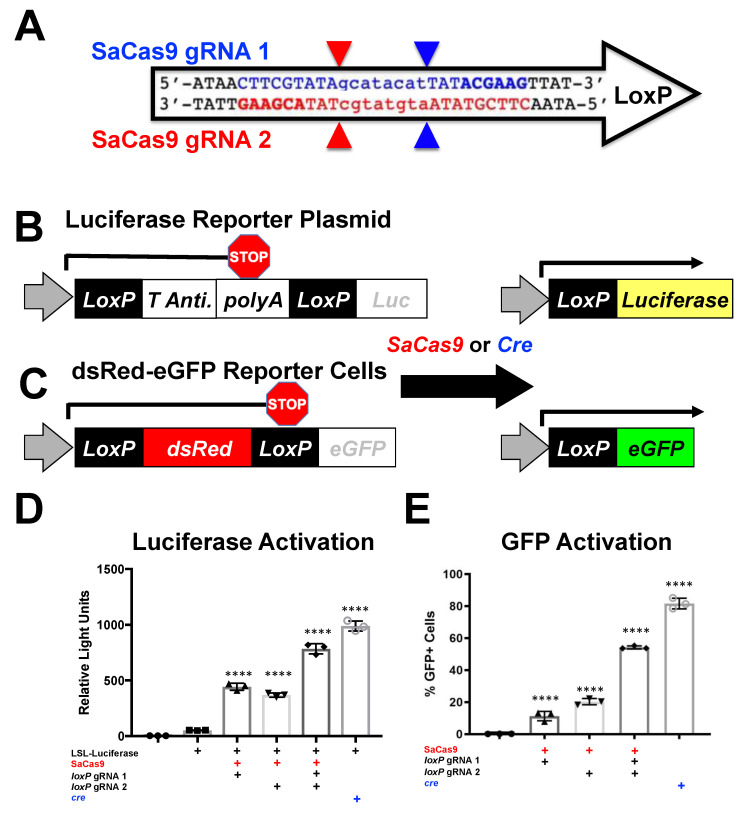
Gene activation through targeting *lox*P in vitro. (**A**) The *lox*P sequence is shown with key features and target sites for CRISPR SaCas9. The capitalized base pairs are the 13-base-pair palindromic regions flanking the 8-base-pair core that gives *lox*P its directionality. The gRNA 1 homologous strand to the guide RNA is depicted in light blue with the PAM-binding region in dark blue and bold letters and a triangle to indicate the cleavage site. gRNA 2 depicts the same in red. (**B**) This depicts the reporter plasmid p133, the LSL-Luc reporter plasmid used in D, and the general outcome when treated with SaCas9 with targeting *lox*P gRNAs or cre recombinase. (**C**) The lentiviral vector is used to make the Red/Green reporter cells used in (**E**) and similarly depicts the outcome when gene edited by SaCas9 or Cre. (**D**) Cells were plated into a 6-well plate and transfected at 60–80% confluency with Xfect and 2.5 μg of the reporter plasmid was transfected to be targeted by cre or SaCas9. Groups were compared by one-way Anova and Tukey’s multiple comparison. Using this, all the groups were significant compared to other groups except between the untransfected control group and the P133 transfected group and between the individual gRNA treated groups; 95% confidence intervals are shown and the significance against the control is shown (n = 3). (**E**) The Red/Green HEK293 reporter cells were transfected with 5 μg of plasmid. The single gRNA groups were significant compared to untransfected and the combination of gRNAs and cre was significant compared to all groups by one-way ANOVA with Tukey’s multiple comparisons. Individual gRNAs were not significant compared to each other (n = 3) (**** *p* < 0.00001).

The Barry lab previously made use of these floxed reporter mice to “fingerprint” gene delivery in vivo by adeno-associated virus (AAV) vectors [10]. In this approach, AAV-cre was injected into mice that are transgenic with different cre-activated reporter genes. In LSL-luciferase mice, luciferase’s expression is blocked by a floxed polyA cassette upstream of luciferase (Figure 1). In the absence cre, no luciferase is expressed. When cre is delivered, the recombination process results in the net deletion of the floxed stop cassette consisting of neomycin and a polyA transcriptional stop, resulting in an activated luciferase reporter. In mT/mG mice, a floxed membrane-targeted red fluorescent protein mTomato (mT) is followed by membrane-targeted GFP (mG). In the absence of cre, mT is expressed in all cells of the mouse and is membrane targeted. When cre is delivered, mT is deleted and mG is expressed. At higher magnifications, these membrane-targeted reporter proteins provide substantial cell discrimination [8].

More recently, a similar “footprinting” system was used to track gene delivery with cre recombinase and notably Cas9 by monitoring fluorescent protein activation [11]. This approach lacked the ability to track editing in living animals by luciferase imaging but had the added value of tracking Cas9 in vivo post-mortem by tissue sectioning. In this case, Cas9 was targeted by gRNA to a polyA “stop” cassette near *lox*P sites in mice bearing floxed inactivated fluorescent protein genes. This design is limited to the specific stop cassette in this mouse model and is not widely applicable to other mouse reporter models. Cas9 cleavage followed by host cell DNA repair was able to activate reporter gene expression to monitor in vivo editing [11].

While this footprinting approach was novel, its targeting of the polyA sequence will work only in a small set of 10 fluorescent and neural mouse models that have the same stop cassette [12]. Mice that have stop cassettes that express a gene rather than repeated polyAs as the stop cassette, like the LSL-luciferase and mT/mG mice (see Graphical Abstract), cannot use a polyA based gRNA. This is because the gRNAs will not create a large deletion and can even have off-target editing of the similar target sites. Additionally, if making a hybrid mouse with 2 different *lox*P cassettes, the only site guaranteed to be an editable target on both chromosomes would be *lox*P.

Given this, CRISPR-Cas9 was targeted to *lox*P sequences and tested in the combined luciferase and mT/mG mouse model for combined live imaging and post-mortem evaluation of genome editing. By doing this, it was shown that SaCas9 activation of these reporter genes has near equivalent activity to that of cre recombinase, in vitro and in vivo. Through sequencing, a primary pathway was not conclusively determined due to ambiguity of the repair outcomes, although non-homologous end-joining events were amongst the top reads. Finally, the targeting of *lox*P with SaCas9 and other CRISPR nucleases has opened the door to using the plethora of established *lox*P models to test new delivery methods and monitoring for CRISPR gene editing activity in vivo.

## 2. Materials and Methods

### 2.1. Plasmids and Cloning 

The reporter plasmid p133 pSV-STOP-luciferase was a gift from Jeffrey Green (Addgene plasmid #8390; http://n2t.net/addgene:8390; RRID: Addgene_8390, accessed on 15 April 2020) and consisted of an SV40 promoter upstream of a floxed set of SV40 polyA signals with firefly luciferase downstream of the floxed region (Addgene, Watertown, MA, USA). Plasmid px601 was purchased from Addgene and consisted of a CMV-expressed SaCas9 and gRNA expression cassette flanked by AAV ITRs (pX601-AAV-CMV::NLS-SaCas9-NLS-3xHA-bGHpA;U6::BsaI-sgRNA was a gift from Feng Zhang (Addgene plasmid #61591; http://n2t.net/addgene:61591, accessed on 15 April 2020; RRID: Addgene_61591)). Annealed oligos were inserted into the gRNA cassette after plasmid px601 was digested with BsmBI. This method was previously described by Ran et al. [13]. The plasmids that were successfully cloned with the *lox*P gRNAs 1 and 2 and were named plasmid px601 *lox*P1 and plasmid px601 *lox*P2, respectively. pSC-CMV-cre was previously developed by the Barry lab and consisted of a CMV-driven cre flanked by AAV ITRs [10]. ErCas12a plasmid was cloned as previously described in Wiersen et al. [14]. 

### 2.2. Cell Culture 

HEK293 cells purchased from ATCC (ATCC^®^ CRL-1573™) were grown with Dulbecco’s modified Eagle’s medium completed with 10% fetal bovine serum (Thermo Fisher Scientific, Waltham, Massachusetts, USA) and penicillin–streptomycin at 100U/mL (Thermo Fisher Scientific). Plasmid transfections were done with Xfect Transfection Reagent from Takara Bio into 6-well plates of HEK293 cells when the cells were at 70% confluency following manufacturer’s instructions. Transfections consisted of 2.5 µg of editing plasmids (px601 gRNA1, px601 gRNA2, and pSC-CMV-cre) and 2.5 µg of reporter plasmids. Transfections that used two gRNAs used 1.25 µg of px601 *lox*P1 and px601 *lox*P2 each. Cells were harvested 48 h post-transfection for luciferase assays. HEK293 cells were transduced with a lentivirus constructed from pLV-CMV-*lox*P-DsRed-*lox*P-eGFP and selected for using the puromycin selection marker. pLV-CMV-LoxP-DsRed-LoxP-eGFP was a gift from Jacco van Rheenen (Addgene plasmid # 65726; http://n2t.net/addgene:65726, accessed on 15 April 2020; RRID:Addgene_65726) [15]. Once these were established, the cells acted as the reporter system in Figure 1C and had the activity shown in Figure 1E (the IRES puromycin selection cassette downstream of the fluorescent cassettes is not shown). The cells were transfected with 5 μg of px601 *lox*P1, 5 μg of px601 *lox*P2, and 2.5 μg of each or with 5 μg of cre reporter plasmid. For the ErCas12a analysis, cells were plated into a 6-well plate and transfected at 60–80% confluency with Xfect and 2.5 μg of the reporter plasmid was transfected to be targeted by ErCas12a. The ErCas12a plasmid co-expressed either gRNA 1 or 2 and was co-transfected at 2.5 μg. For the cells transfected with both gRNAs, 1.25 μg of each was transfected into that well. Cells were harvested for a luciferase assay. By one-way ANOVA, all the groups were significant except between the untransfected control group and the P133-transfected group and between the individual gRNA-treated groups; 95% confidence intervals are shown (*n* = 3).

### 2.3. Luciferase Assay 

Cells were harvested from each condition via trypsin. The cells were spun down at 500× *g* for 10 min and the supernatant was removed. The cells were resuspended in 400 µL of PBS and pipetted vigorously to have a homogenous mixture of cells. Then, 100 µL of cells was pipetted into a clear bottom, black, 96- well plate. This was done in triplicate for each group of cells and 100 µL of room temperature Bright-Glo was then applied to each well. Wells were then analyzed via the BioTek Synergy H1 Microplate Read and the BioTek Gen5 Microplate Software.

### 2.4. Flow Cytometry 

Cells were harvested as with the luciferase assay. The cells were passed through a 100 μm filter to avoid clumping of cells. The cells were then assayed by the Microscopy and Cell Analysis Core at Mayo Clinic Rochester (Rochester, Minnesota, USA) using a BD FACSCanto. The data were then processed via FloJo.

### 2.5. Mice 

LSL-luciferase mice (a.k.a. FVB.129S6(B6)-GT(ROSA)26Sor^tm1(Luc)Kael^/J) have a luciferase expression cassette behind a floxed polyA signal domain with the ROSA26 promoter. The mT/mG mice (a.k.a. GT(ROSA)26Sor^tm4(ACTB-tdTomato,-EGFP)Luo^/J mice) have a floxed mTomato behind the pCA promoter with membrane-bound EGFP behind mTomato. All mice were purchased from Jackson Laboratories and all animals were treated according to the provisions of the Animal Welfare Act, PHS Animal Welfare Policy, and NIH Guide. 

### 2.6. Hydrodynamic Injections 

Mice were injected by hydrodynamic tail vein injections [16]. Injections consisted of 25 µg of gene editing plasmids, either px601 *lox*P1 and px601 *lox*P2 mixed in a 50:50 ratio or pSC-CMV-cre. The plasmids were suspended in 2.5 mL (~10% body weight) of PBS and the injection was completed within 8–10 s [16]. Control mice were injected with PBS only. Mice were monitored post-injection for tolerance of hydrodynamic injection. For the dose escalation hydrodynamic injections, 7.5 μg, 25 μg, and 83.3 μg of plasmid were used for the low, medium, and high groups, respectively. Pilot experiments were conducted with intramuscular soleus injections of 100 μg of plasmid in 50 µL of PBS (Supplementary Figure S3).

### 2.7. In Vivo Bioluminescent Imaging 

Mice were anesthetized with 2% isofluorane and maintained at 2% isofluorane. Mice were intraperitoneally injected with 150 µL of 20 mg/mL D-Luciferin (RR Labs, Inc., San Diego, CA, USA). Xenogen IVIS 200 was used to image the mice ten minutes after injection of the D-Luciferin. The dose escalation imaging experiments were done using the IVIS Lumina S5 Imaging System due to the Xenogen being retired. Living Image software was used to quantify the luminescence.

### 2.8. Liver Sectioning and Confocal Microscopy of Mouse Liver 

Livers were harvested and fixed overnight in 4% paraformaldehyde (PFA)-phosphate buffered saline (PBS) at 4 °C. Livers were transferred to 15% sucrose–PBS overnight and then moved to 30% sucrose–PBS at 4 °C until the tissue sunk in the solution to cryoprotect the tissue. Tissues were trimmed and flash frozen in optimal cutting temperature (OCT) medium (Sakura Finetek USA, Torrance, CA, USA). A Leica CM1860 UV cryostat (Leica Biosystems) was used to create cryosections (18 μm thickness) which were mounted on slides (SuperFrost Plus; Thermo Fisher Scientific, Waltham, MA). VECTASHIELD with 4′,6-diamidino-2-phenylindole (DAPI) (Vector Laboratories, Burlingame, CA, USA) was applied with a CytoSeal-60 coverslip sealant (Thermo Fisher Scientific). The confocal microscopy was performed at the Microscopy and Cell Analysis Core facility at Mayo Clinic Rochester (Rochester, MN, USA) using a Zeiss LSM790 laser confocal microscope (Carl Zeiss Jena, Jena, Germany). Representative images were selected from the confocal microscopy and used to count GFP+ cells and total cells.

### 2.9. Sequencing of loxP Junctions 

DNA was isolated from the transfected human 293 cells via the DNeasy Blood and Tissue Kit’s protocol. The primers RVprimer3 (5′-CTAGCAAAATAGGCTGTCCC-3′) and LucNRev (5’-CCTTATGCAGTTGCTCTCC-3′) were used to PCR amplify from the SV40 promoter across the polyA region or the deleted region. Shortening the extension time to 30 s restricts the amplified product to only edited plasmids. This band was then gel-excised and stratacloned to isolate individual sequences. Individual clones were grown up, miniprepped, and sent for Sanger sequencing at Genewiz. Additionally, gel-isolated bands were also sent for next generation sequencing (NGS) via the Genewiz’s Amplicon EZ sequencing using the extended primers RVprimer3 NGS (5′-ACACTCTTTCCCTACACGACGCTCTTCCGATCTCTAGCAAAATAGGCTGTCCC-3′) and LucNRev NGS (5′-GACTGGAGTTCAGACGTGTGCTCTTCCGATCT CCTTATGCAGTTGCTCTCC-3′). Read outcomes were compiled and ranked from most common to least. This method creates ~50,000 reads of the 500bp fragments. This is a biased analysis that will only monitor large deletions of the stop cassette and does not quantify unedited plasmids. These reads were then aligned to the predicted *lox*P regeneration that one would expect from cre recombinase or a one gRNA NHEJ event. This system is set up to monitor mutations at the site of interest and Genewiz sends the reads analyzed with WT, base change, deletion, insertion, and insertion/deletion. WT is the *lox*P regeneration site and this shows the site has recombined into the predicted site for cre recombination with the *lox*P sites being recombined. Base change is a *lox*P regeneration site with a base change within 10bp of the *lox*P site. “Deletion” and “insertion” are as they sound, deletion or insertion, compared to the *lox*P regeneration site and “insertion and deletion” denote reads that show signs of both. The top five outcomes were looked at for potential connection to a DNA repair pathway, but due to the potential for *lox*P regeneration to be the result of any repair pathway, this was not pursued.

### 2.10. Statistical Analysis 

*T*-test and one-way ANOVA with multiple comparisons were done using GraphPad Prism^™^. A 95% confidence interval was displayed on the graphs and those considered significant by one-way ANOVA with Tukey’s multiple comparison were marked as significant. Significance between groups is described in the figure legends in more detail between the groups and beside the control groups.

## 3. Results

### 3.1. CRISPR gRNA Design and In Vitro Evaluation 

The ability to target various Cas enzymes to different sequences is commonly limited by their need to bind specific protospacer adjacent motifs (PAMs) (Table 1) [1,2,14,16,17,18,19,20,21]. *lox*P is a 34-base pair (bp) target sequence in bacteriophage P1 for cre recombinase. The conventional *lox*P sequence ATAACTTCGTATAgcatacatTATACGAAGTTAT has two palindromic 13 bp sequences (upper case) separated by an asymmetric 8 bp “core” sequence (lower case) (Figure 1A). The PAMs of different Cas proteins were evaluated for their ability to target the wild-type *lox*P sequence. For example, the type 1 CRISPR from *Staphylococcus aureus* Cas9 (SaCas9) has a general PAM requirement of NNGRRT where “N” indicates any nucleotide and “R” indicates any purine (A or G) [22]. When this PAM sequence was used to scan *lox*P, it initially appeared that SaCas9 could not target *lox*P. However, the “T” in NNGRR(T) is actually optional [23]. When T was excluded when scanning the site, two different SaCas9 gRNAs (gRNA1 and 2) were identified that target either the top or bottom strand of *lox*P (Figure 1A)**.**

The gRNAs of several other type I and type II CRISPRs were identified as having PAM sequences capable of targeting *lox*P, including ErCas12a, xCas9, AsCas12a RR-Variant, LbCas12a RR-Variant, and AsCas12a RVR Variant (Table 1) [17,18,19,20,21,22,23,24].

### 3.2. loxP-Targeted Genome Editing with SaCas9

Genes can be inactivated by placing a *lox*P-flanked (floxed) polyA stop sequence between the gene’s promoter and its open reading frame. cre recombinase can activate expression from such a gene by deleting the stop sequence.

In theory, if SaCas9 can cleave both *lox*P flanking the polyA sequence, the host cell may be able to repair these breaks resulting in deletion of the stop sequence. If so, we hypothesized that SaCas9 might be able to activate luciferase expression in a manner like cre recombinase to delete the same stop polyA sequence (Figure 1B). 

To test this, SaCas9 gRNA1 and 2 were cloned separately into plasmids expressing the SaCas9 and a gRNA. These plasmids were transfected into HEK293 cells with a plasmid p133 that has a floxed SV40 polyA signal upstream of the firefly luciferase cDNA (Figure 1B). Under these conditions, both single gRNAs enabled SaCas9 to activate luciferase expression (Figure 1D). When the two gRNAs were co-transfected together with the luciferase plasmid, reporter gene expression increased nearly two-fold. This level of gene activation was notable as it approached the level of luciferase activation that was mediated by cre recombinase itself. 

To test the editing efficiency, dsRed/eGFP stable 293 cells were created using the pLV-CMV-*lox*P-DsRed-*lox*P-eGFP (Figure 1C). These cells were then transfected with px601 *lox*P1, px601 *lox*P2, a combination of the two gRNA-SaCas9 plasmids or cre plasmid. (Figure 1E). These cells were then assayed by flow cytometry. These data show a similar trend to the luciferase assay.

**Figure 2:**
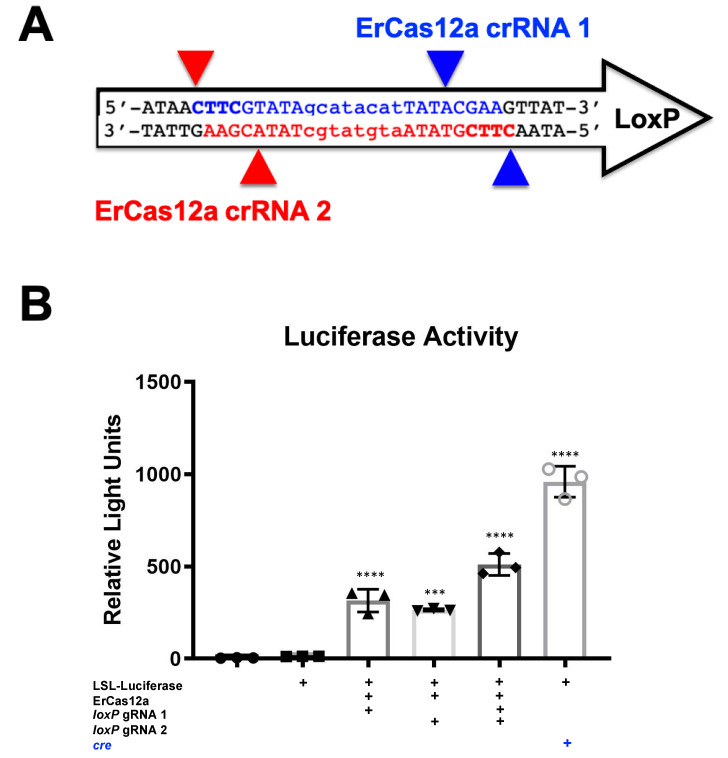
ErCas12a-mediated gene activation through targeting *lox*P in vitro. (**A**) The *lox*P sequence is shown with key features and target sites for CRISPR ErCas12a. The capitalized base pairs are the 13-base-pair palindromic regions flanking the 8-base-pair core that gives *lox*P its directionality. gRNA 1 homologous strand to the guide RNA is depicted in light blue with the PAM-binding region in dark blue and bold letters and a triangle to indicate the cleavage site. gRNA 2 depicts the same in red. (**B**) The ErCas12a plasmid co-expressed either gRNA 1 or 2 and was co-transfected with the LSL-Luc reporter plasmid at 2.5 ug. By one-way ANOVA with Tukey’s multiple comparisons, all the groups were significant except between the untransfected control groups (untransfected versus reporter plasmid only) and between the individual gRNA-treated groups (*lox*P gRNA 1 vs. *lox*P gRNA 2); 95% confidence intervals are shown (n = 3) (*** *p* < 0.0005 and **** *p* < 0.0001).

To determine if other genome editors (Table 1) might also mediate this effect, ErCas12a plasmids were also transfected along with p133 reporter plasmid. Under these conditions, both were able to induce luciferase activity, albeit at lower levels than SaCas9 (Figure 2).

### 3.3. Host Cell Repair of SaCas9-Cleaved loxP Sites

**Figure 3:**
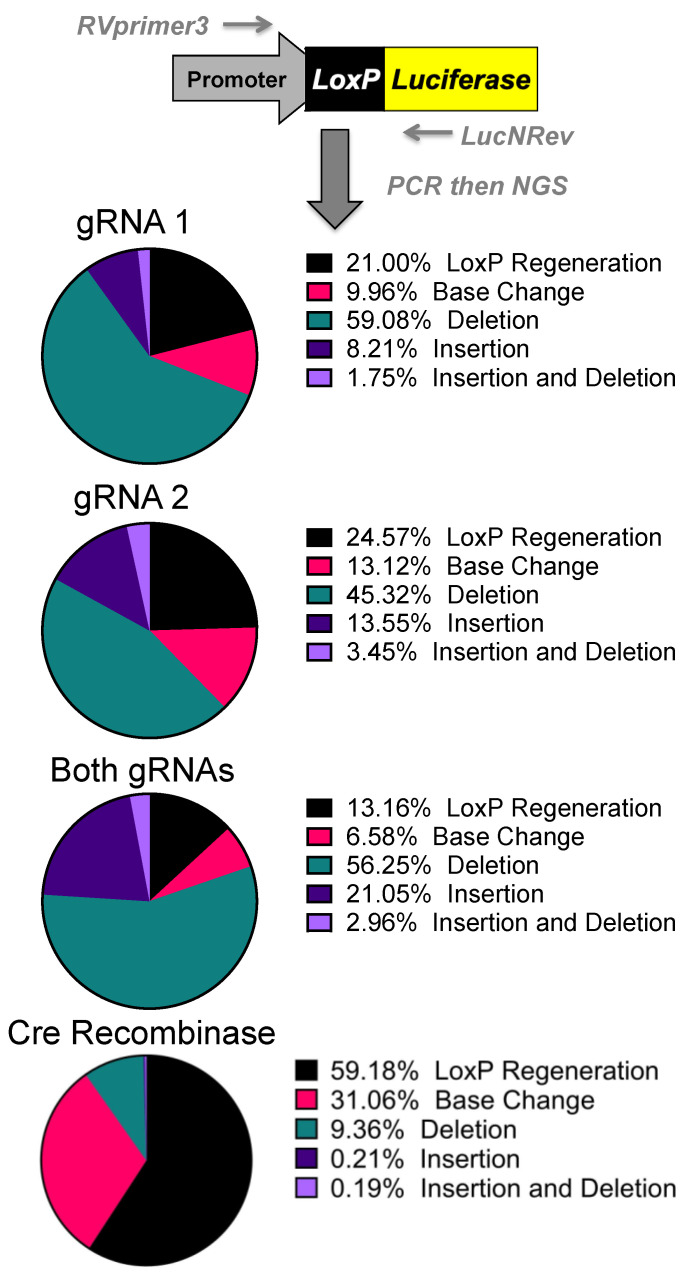
Sequencing of Repair Juncture. DNA was harvested from the previous transfections and amplified using primers in the SV40 promoter and luciferase with next generation sequencing (NGS) sequencing adapters. Using Amplicon-EZ through Genewiz, the deletion results were analyzed and quantified (n = 1).

DNA was purified from cells that were transfected with the p133 luciferase reporter and gene editing plasmids. A 500-base-pair fragment containing the *lox*P sites was PCR amplified and assessed by next generation sequencing. These sequences were aligned against the floxed sequence, and the outcomes of recombination or DNA repair were evaluated (Figure 3 and Supplementary Figure S1). When cre-modified fragments were evaluated, more than half accurately deleted the polyA sites and regenerated an intact *lox*P site (Figure 3). Interestingly, nearly half of the sites were inaccurately recombined by *lox*P generating mutations, deletions, or insertions. The same evaluation of SaCas9-treated fragments revealed markedly lower regeneration of the wild type *lox*P sequence.

About a quarter of the reactions mediated by single gRNA1 or gRNA2 generated an intact *lox*P and the rest were a variety of mutations. When both gRNAs were used, this reduced regeneration of *lox*P to only 13% of outcomes. Analysis of the specific repair junctions suggested that most were resolved by non-homologous end-joining rather than by homology-directed repair or microhomology-mediated repair (Figure 3 and Supplementary Figure S1).

### 3.4. Monitoring In Vivo Genome Editing in Living Animals

**Figure 4:**
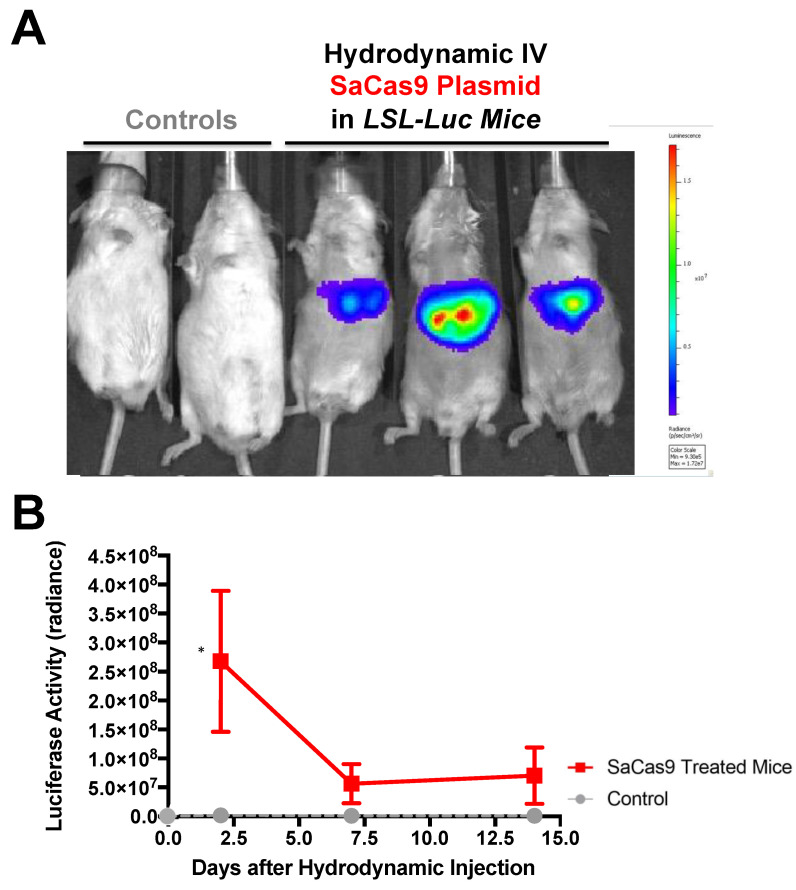
Hydrodynamic delivery in luciferase reporter mice. (**A**) LSL mice contain a floxed stop cassette blocking luciferase expression. Gene editing by CRISPR gene editing excises the stop cassette exposing luciferase to the promoter allowing for expression. LSL mice were injected hydrodynamically with SaCas9 gene editing plasmids. Control mice received a 2.5 mL PBS injection and the SaCas9 plasmid mice received 12.5 μg of each plasmid in the 2.5 mL of PBS. (**B**) Mice were monitored for luciferase activity on days 2, 7, and 14. Using a *t*-test for each day, the SaCas9-treated groups were significant compared to the control on Day 2; 95% confidence intervals are shown (n = 3) (* *p* < 0.05).

To determine if SaCas9 with gRNAs targeting *lox*P would be effective in vivo, *lox*P-Stop-*lox*P luciferase (LSL-luciferase) mice with a *lox*P-inactivated luciferase gene were injected by hydrodynamic intravenous injection with the SaCas9 gRNA1 and gRNA2 plasmids and the animals were imaged for luciferase activity up to 14 days after injection (Figure 4). 

Under these conditions, the Cas9 and gRNA plasmids appeared to activate the genome copies of luciferase as evidenced by the generation of photons of light from the liver (Figure 4A). Sustained luciferase activity was observed for over 2 weeks after a single SaCas9 and gRNA plasmid injection (Figure 4B). 

**Figure 5:**
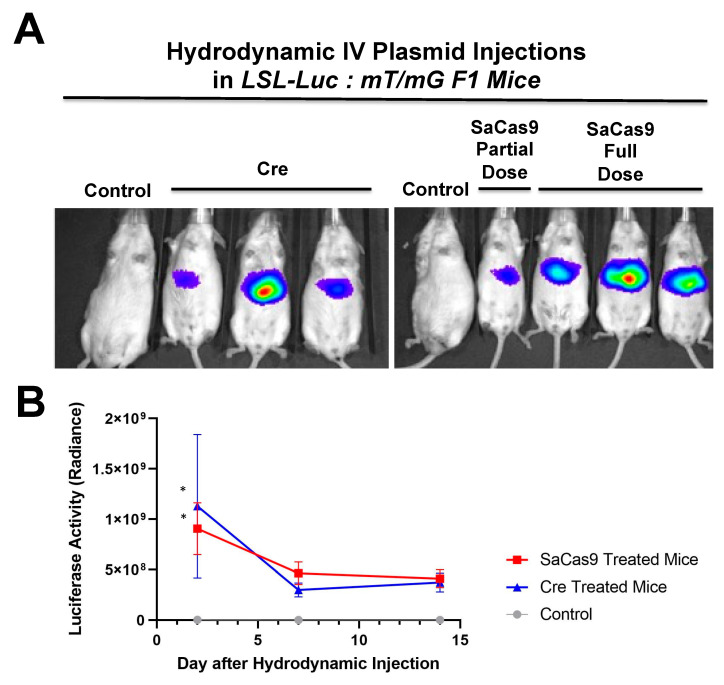
Hydrodynamic delivery in hybrid reporter mice. (**A**) LSL mice were crossed with RG mice to produce a crossed reporter mouse. These mice are sensitive to cre recombinase or CRISPR activity leading to luciferase expression and GFP expression. Mice were hydrodynamically injected with cre recombinase or SaCas9 expressing plasmids. Control mice received a 2.5 mL PBS injection, the SaCas9 plasmid mice received 12.5 μg of each plasmid in the 2.5 mL of PBS, and the cre groups received 25 μg of cre plasmid in 2.5 mL of PBS. (**B**) Luciferase activity was monitored within the mice on days 2, 7, and 14 and *t*-tests were carried out. Comparing via *t*-tests showed that the editing groups were significant on Day 2 compared to the control. The lines denote a 95% confidence interval (n = 3) (* *p* < 0.05).

Hybrid mice generated from crossing LSL-luciferase mice with mT/mG mice have exactly one gene copy of cre-activatable luciferase and exactly one gene copy of the mT/mG cassette (Figure 5 and Graphical Abstract). Therefore, these hybrid mice provide an “on/off” system to detect vector transduction and pharmacodynamics. The presence of three reporter genes enables 1) in vivo imaging, 2) cell-specific transduction monitoring via mG expression, and 3) on/off confirmation of transduction by coordinated loss of mT with activation of mG [12]. This system can also be applied to the vast repertoire of mice engineered for tissue-specific cre expression (https://www.jax.org/research-and-faculty/resources/cre-repository, accessed on 9 April 2020).

SaCas9 activity was next compared to cre recombinase in LSL-Luc:mT/mG hybrid mice (See Graphical Abstract). Mice were hydrodynamically injected with cre plasmids or a combination of Cas9 gRNA1 and 2 plasmids and luciferase imaging was performed as in Figure 4.

Luciferase activity generated by the introduction of SaCas9 was like that generated by injection of cre recombinase plasmid (Figure 5). Following this live animal imaging, the animals were sacrificed, and their livers were sectioned for confocal microscopy to observe mT and mG at cellular resolution.

**Figure 6:**
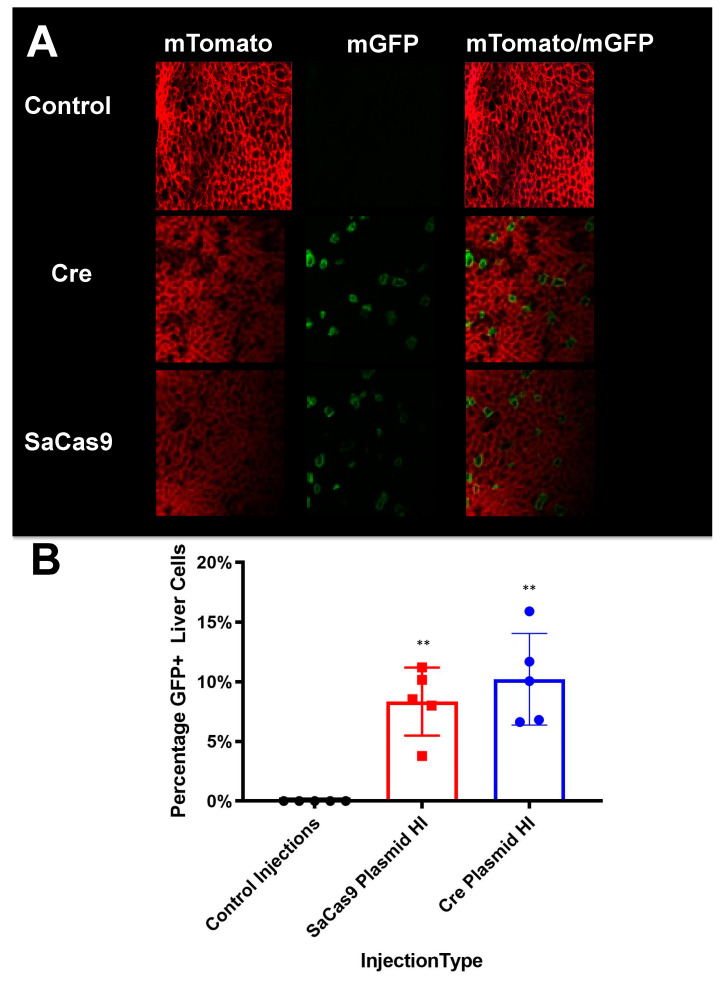
Liver sectioning from hybrid mice. (**A**) Mouse livers were harvested, fixed, and imaged for tdTomato and GFP activity. Representative microscopy shows cre- and SaCas9-treated mouse livers. (**B**) Converted cells were counted as a percentage of total cells in representative images across the gene-edited mice. The bars are 95% confidence intervals and were found significant compared to the control by one-way ANOVA and Tukey’s multiple comparisons. The edited groups were not significant compared to each other (n = 5) (** *p* < 0.005).

These analyses showed that cre and SaCas9 plasmids mediated conversion of cells from mTomato to mGFP expression in a subset of cells (Figure 6A). When GFP-positive cells were counted on representative slides this indicated that SaCas9 and cre mediated similar conversion of approximately 10% of cells from mT to mG expression after hydrodynamic injection of these plasmids (Figure 6B).

**Figure 7:**
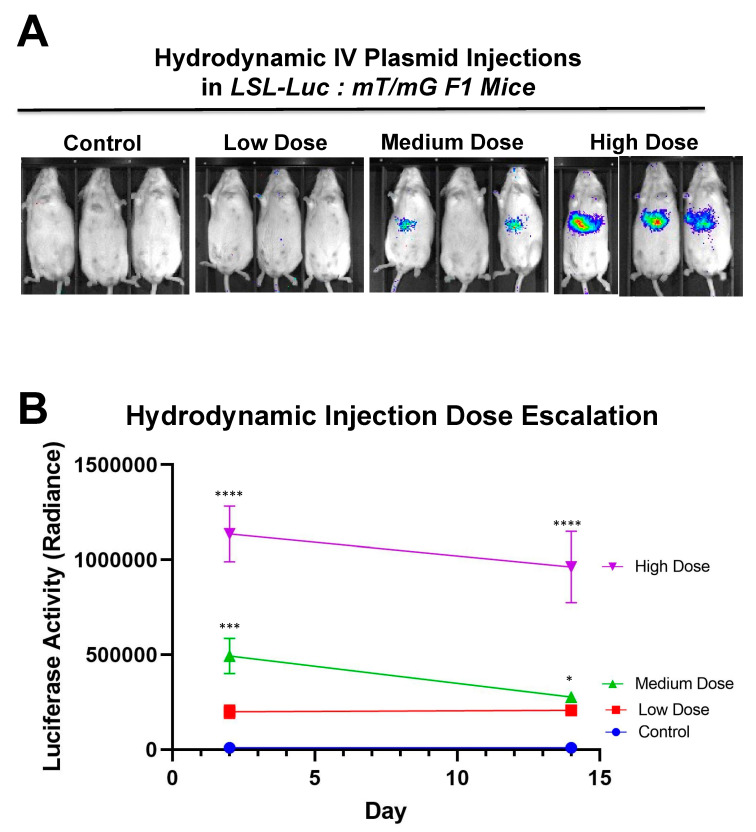
Hydrodynamic dose variation injections. (**A**) Mice were hydrodynamically injected at varying doses of 7.5 μg, 25 μg, and 83.3 μg of plasmid. Day 2 mouse images are shown. (**B**) Radiance levels emitting from the mouse livers were measured and compared. Each day was analyzed by one-way ANOVA and Tukey’s multiple comparison. High and medium doses were significant compared to the control on both days while low dose was not significant compared to the control on either day via one-way ANOVA with multiple comparison. Via one-way ANOVA, high, medium, and low doses were significant compared to each other on Day 2. On Day 14 and by one-way ANOVA with Tukey’s multiple comparisons high dose was significant compared to all other groups. Medium dose was only significant compared to the control dose on Day 14. All other comparisons on Day 14 were not significant by one-way ANOVA with Tukey’s multiple comparisons (n = 3) (* *p* < 0.05, *** *p* < 0.0005, **** *p* < 0.0001).

Additionally, to determine the system’s ability to have a dose-based response, mice were hydrodynamically injected with varying doses of SaCas9 plasmid (Figure 7A). This shows that the dose of SaCas9 does affect the luciferase reporter activity as seen by the varying activity levels measured (Figure 7B). Mice were shown to have a dose response with significance between the high dose and medium dose vs. the lower doses and the high dose vs. the medium dose by one-way ANOVA with multiple comparison.

## 4. Discussion

This study was performed to enable a facile system for monitoring genome editing in living animals as well as to identify edited tissue at a cellular resolution. This work shows that established cre-*lox*P reporter systems can be used to monitor CRISPR activity. This paper showed that *lox*P cleavage by SaCas9 can be comparable to cre recombinase activity in deleting stop signals in vitro or in vivo. By using the previously established mT/mG:LSL Luc, it was demonstrated that CRISPR activity could be detected non-invasively in living mice along with a quantitative approach upon dissection of edited tissues. 

This system also establishes the first *lox*P specific reporter for fluorescence monitoring. Previous work has used gRNAs to target adjacent to the *lox*P site or within the polyA region (Supplementary Figure S2A). While these have shown the ability to monitor CRISPR activity via tissue sectioning and DNA analysis, these systems are specific to Ai9 mice and a few related strains and would not be broadly applicable to other reporter systems such as the LSL-mice or the mT/mG target loci [25]. Other work has shown in vitro targeting with partial overlap with *lox*P and complete coverage with the gRNA in the *lox*P mutants *lox71* and *lox*66 (Supplementary Figure S2B) [26]. These previous gRNAs would not be applicable to most mouse models that likely will not have convenient adjacent sequences that allow for targeting and most mouse reporter models do not use *lox*P mutants. The system shown herein can be used with any wildtype LoxP site and is therefore capable of being used with any mouse model that uses *lox*P to activate or deactivate genes by cre excision. This system could also be used in cases where *lox*P is positioned for gene inversion to delete a region or disrupt this process.

Cre recombinase has evolved to not only cut *lox*P sequences, but to also repair the resulting genomic lesion. In contrast, Cas editors simply cleave DNA. Any Cas cleavage event must rely on the host cell to repair the cut DNA. Therefore, one might assume that Cas-mediated deletion of a floxed locus would likely be less efficient than the evolved cut and repair cre recombinase system. However, when using SaCas9 and the dual guide system, SaCas9 was like cre in efficiency for reporter activation in vitro and in vivo (Figure 1, Figure 4 and Figure 5). This activity level can be partially explained by SaCas9, an efficient DNA editor that can show higher activity than SpCas9 and a Cas12a variant when using similar or identical targets [21].

Hydrodynamic delivery functions by rupturing the cell membranes under pressure, pushing the plasmid DNA into the cell. This pressure and resulting damage could be responsible for the decline in luciferase activity from Day 2 to Day 7 as seen in Figure 3, Figure 4 and Figure 6. This coupled with the introduction of neo-antigens in the form of SaCas9 or cre could lead to immunological responses to transduced cells as well. Additionally, there is a discrepancy between the SaCas9 hydrodynamically injected mice in Figure 4; Figure 5. Older mice are not as effective for hydrodynamic delivery and older mice were used in Figure 4. Different maxi-preps were used between the experiments so this may have also contributed.

Additionally, Figure 7 shows that there is a dose response to differing amounts of SaCas9. This is of value because it shows that this system could be used to monitor variant levels of delivery via luciferase activity. Whether SaCas9 is delivered by nanoparticle, virus, ribonucleoprotein, or some novel method, this provides a method to detect variations in delivery efficiency non-invasively and with cellular specificity. It should also be noted that the luciferase levels showed a significant decrease compared to previous experiments. We believe this to be the result of imaging differences between the Xenogen and the Lumina. Supplementary Figure S3 also shows that this reporter system works in tissues beyond the liver. The Barry lab has also previously published in Hillestad et al. that they were able to detect cre recombinase activity using this reporter mouse in the liver, heart, lungs, muscles, brain, kidney, and spleen [10].

Next generation sequencing analyses of the gene editing outcomes provide some insight into how the cell repairs these DNA breaks prior to reporter activation. Three repair pathways were non-homology-induced repairs, small local homology (microhomology), and homology-based outcomes, especially considering that deletions occur between two identical *lox*P sequences. Analyses of deletions induced by individual gRNAs demonstrated that most of the edits result in the recreation of a *lox*P site. Unfortunately, these can be the result of either NHEJ or homology-based repair when using individual gRNAs (Supplementary Figure S1).

Analysis of the dual gRNA-induced deletions demonstrated a different mix of outcomes (Supplementary Figure S1). After the repair pathway-ambiguous *lox*P recreation, the next two top reads consisted of NHEJ-based events that are caused by different gRNAs targeting each *lox*P site. Although the top reads are ambiguous, the top reads that are capable of being definitively linked to a pathway are NHEJ. Depending on which gRNA edits which *lox*P site will determine whether an 8bp region would be duplicated or deleted. That being said, these cuts have the potential to cause microhomology-based events, only one of which is distinguishable as such. The strong prevalence of NHEJ-specific events strongly suggests that this is a major repair mechanism, but with the top detected outcome being ambiguous and repeated cutting of regenerated *lox*P sites confusing the data further, it cannot be determined at this time what is the primary repair pathway. Further parsing of the mechanism may be done in future work by specifically knocking down proteins related to these pathways.

The sequencing was initially done to explore the possibility of alternative DNA repair pathways being responsible for the increased efficacy of both gRNAs over individual gRNAs. While repair mechanisms cannot be determined conclusively, the increases in efficacy seen with both gRNAs are potentially connected with CRISPR gene editing selecting for mutations that prevent further cutting. With a single gRNA, there is the potential for the gRNA target site to be cut and result in an insertion or deletion (indel) rather than result in a large deletion and preclude the possibility for a subsequent DSB generating a large deletion. An indel near the cut site of the gRNA target site would greatly reduce, if not inhibit, SaCas9 cutting as seen by some of the top reads in NGS [27]. If a *lox*P site is mutated to prevent CRISPR cutting at both *lox*P sites, large deletion of the stop cassette would be prevented. With two potential gRNA targets, the potential indel would be further away from the PAM and less likely to inhibit SaCas9 binding [28]. This may potentially explain the increased efficacy seen with two gRNAs rather than one.

By targeting Cas9 to the *lox*P site, there is also the opportunity to enable further manipulation of these sites. These models can be used in conjunction with targeted insertion technology to deliver genes of interest at *lox*P sites. This could be done to modify the sites to express a different gene or to reconstitute stop cassettes with mutant *lox*P sites that are resistant to CRISPR cutting but available to cre recombinase. There is also the potential of creating a three-outcome cassette: starting cassette, cre recombinase-treated expression cassette, and CRISPR-treated cassette. This would give greater control over animal models that would be able to turn on defective genes via cre recombinase and then deactivate them by CRISPR. There is also the potential of using the deactivated CRISPR enzyme as an inhibitor of cre recombinase, binding *lox*P and preventing binding and recombination. This could act to prevent cre recombinase activity in specific cell populations.

It should be noted that despite the great activity shown by SaCas9 targeting *lox*P, results may vary depending on the DNA site being targeted or the CRISPR being used. This SaCas9 reporter system is highly efficient while the ErCas12a has lower activity. (Figure 1 and Figure 2). This can be attributed, at least partially, by differential binding and cleavage kinetics demonstrated by Cas12a type effectors compared to Cas9. It would be of interest in the future to determine if similar Cas12a effectors such as AsCas12a or LbCas12a can improve upon this foundational work with SaCas9. It is important to note that this may overestimate the activity when using a gRNA relevant to a gene therapy application or a CRISPR other than SaCas9.

This three-way reporter system can be applied to in vivo delivery of CRISPR systems to assess the tropism of the delivery system on a broad and narrow level along with a timeline of CRISPR editing. More broadly speaking, this system can be used in combination with any *lox*P system that relies on deletion for its activity, for which there are over 3000 mice on JAX Laboratories website related to the cre-*lox* system.

## Figures and Tables

**Table 1:** List of CRISPRs with compatible PAMs that enable targeting within a conventional *lox*P.

CRISPR Variants	PAM Sequence	CRISPR Class
SaCas9	NNGRRT	I
ErCas12a	YTTN	II
xCas9	NG, GAA	I
SpCas9-NG (SpG)	NGN	I
SpRY	NRN>NYN	I
AsCpf1 RR-Variant	TYCV	II
LbCpf1 RR-Variant	TYCV	II
AsCpf1 RVR-Variant	TATV	II

## Data Availability

Data will be available online at MDPI Genes and available for download.

## Supplementary Materials

The following are available online at https://www.mdpi.com/article/10.3390/genes12081204/s1, Figure S1: Top 5 Next Generation Sequencing Outcomes, Figure S2: Prior loxP Adjacent gRNAs, Figure S3: Intramuscular Pilot study Injection.
