# Supplementary material for: Unlocking loxP to Track Genome Editing In Vivo
Source: Genes (Basel). 2021 Aug 3;12(8):1204. doi: 10.3390/genes12081204 (PMC8394901; doi:10.3390/genes12081204)
Supplement: Supplementary file 1 [file genes-12-01204-s001.zip › genes-1242336-SI.pdf]

genes

MDPI

Article

Unlocking LoxP to Track Genome Editing *In Vivo*

William A.C. Gendron[1], Jeffrey D. Rubin[1], Michael Hansen[1], Rebecca Nace[1], Brandon W. Simone[2], Stephen C. Ekker[2] and Michael A. Barry[3]*

Supplementary Materials

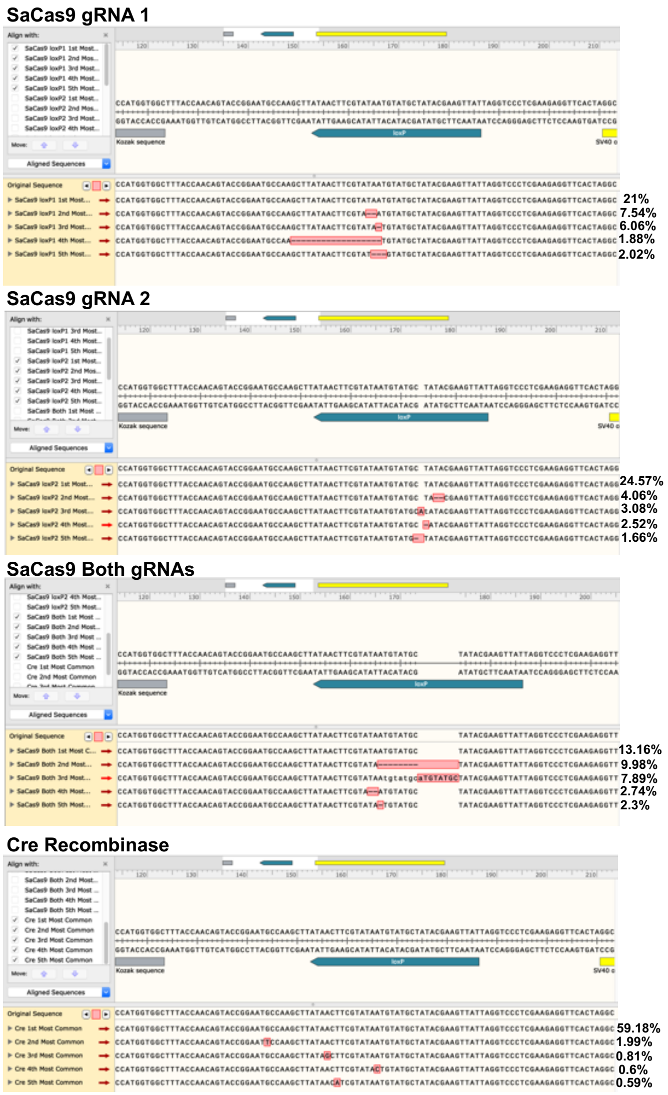

Figure S1. Top 5 Next Generation Sequencing Outcomes: This figure depicts the top 5 repair outcomes detected from next generation sequencing of the luciferase reporter plasmid when co-transfected witb SaCas9 gRNA 1, SaCas9 gRNA 2, a mix of both or Cre Recombinase.

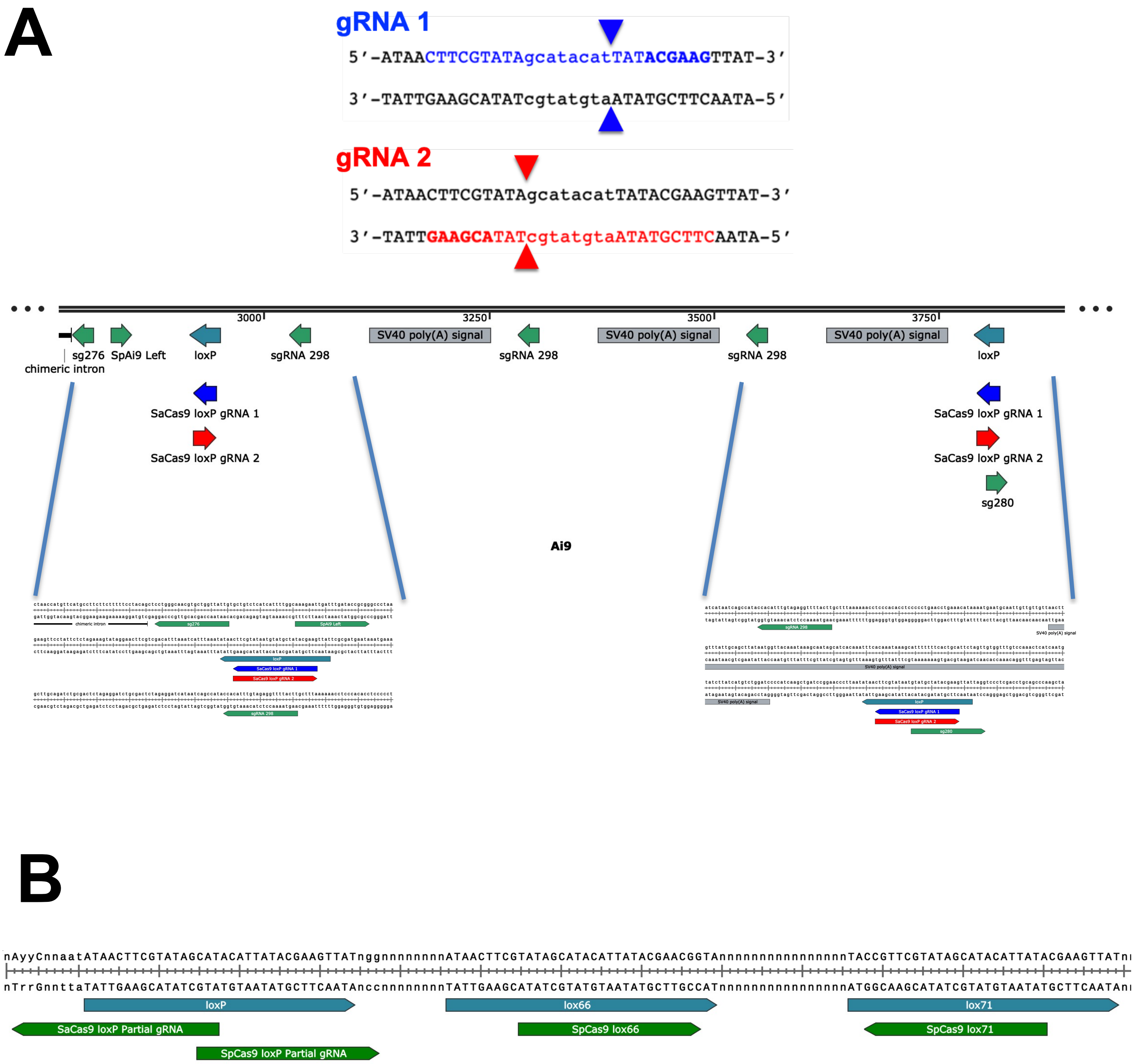

Figure S2. A) Prior Examples of Reporter gRNAs: Previous papers have described using "loxP"**.** Targeting gRNAs. This depicts the closest previously used gRNAs in green used to target the loxP site inside a mouse model. These gRNAs are either adjacent or only partially fall within the loxP sites restricting their use to the specific Ai9 mouse model. While gRNAs adjacent to the loxP sites were tested, the gRNAs used in similar work target within the poly(A) region of Ai9 mice making these gRNAs specific to this model and not widely applicable to loxP models. sg298 was the primary gRNA used by work monitoring Ai9 editing although other gRNAs were tested. In red and blue, the SaCas9 gRNAs developed in this paper are shown. B) This depicts other prior examples of *lox*P gRNAs that either rely on some additional sequence outside of the wildtype *lox*P and specific gRNAs for the mutant *lox*P sites, *lox71* and *lox66*.

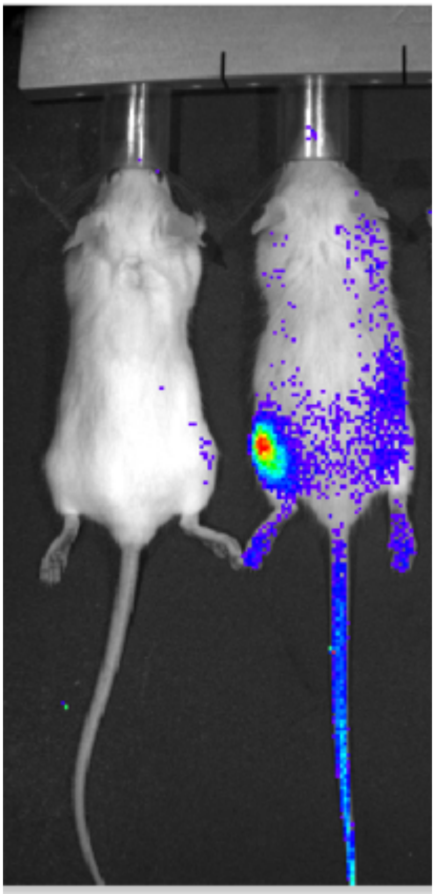

Figure S3. Pilot study showing an intramuscular injection into the left leg of the second mouse showed positive activity from the injection. 50ul PBS with 50ug of each SaCas9 plasmid was injected. Mice were imaged on day 2.
